# Wreaking Reproductive Havoc One Chemical at a Time

**DOI:** 10.1371/journal.pbio.2000706

**Published:** 2016-08-24

**Authors:** Liza Gross

**Affiliations:** Public Library of Science, San Francisco, California, United States of America

In the 1930s, biochemist Edward Charles Dodds hoped to find synthetic estrogens that could treat gynecological ailments caused by hormone deficiencies. In 1936, Dodds identified one candidate that stimulated the female reproductive system in rats whose ovaries had been removed. But the estrogenic activity of the compound, bisphenol A, paled next to the much stronger estrogen he would soon develop himself: diethylstilbestrol (DES).

Dodds had noticed estrogen’s similarity to cancer-causing substances in early studies, but assumed that any drugs would be used only as short-term therapies, so the cancer risk would be small. He never expected DES or any other estrogens to be given to healthy women. Yet some 4 million expectant mothers received DES to prevent miscarriage before physicians realized they’d made a terrible mistake. DES greatly increased the risk of rare vaginal and cervical cancers in exposed daughters. Sons had a higher risk of testicular growths.

**Fig 1:**
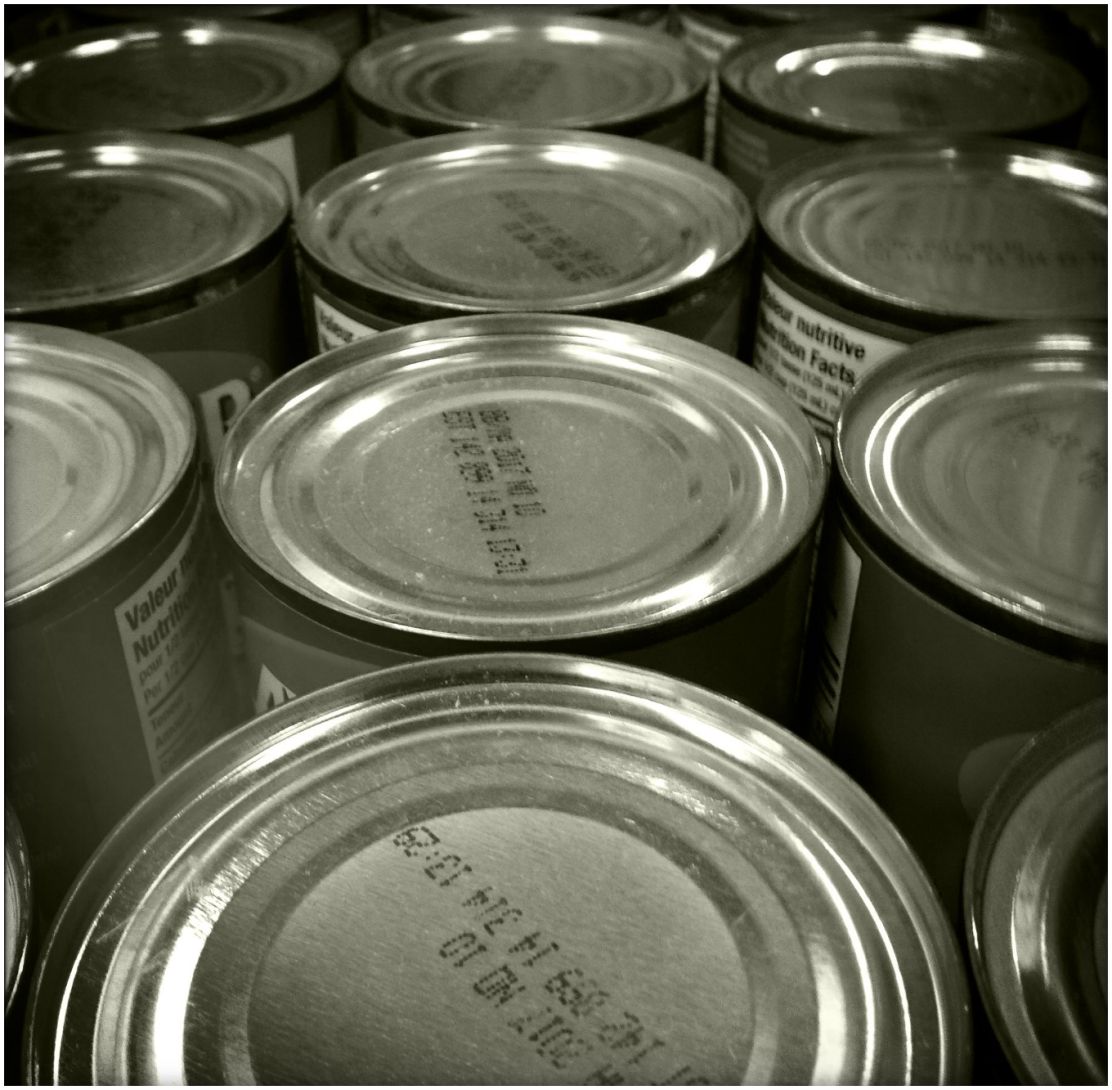
Canned foods are considered one of the most significant routes of human exposure to bisphenol A (BPA). Image credit: elenghan, Flickr.

Bisphenol A (BPA), unlike DES, remained obscure until the 1950s, when chemists tapped it to make polycarbonate plastics and epoxy resins. BPA now tops the list of high-volume chemicals, and is found in numerous consumer products, including water bottles, food packaging containers and can linings, and thermal paper products like cash receipts and boarding passes (Fig 1). And because it can leach out of products, it’s been detected in the urine of nearly every person tested. It’s also been found in breast milk, follicular and amniotic fluid, cord blood, placental tissue, fetal livers, and the blood of pregnant women.

Hundreds of studies have associated the BPA levels found in most of us with reproductive disorders, cancers, obesity, and other adverse effects in both animals and humans. Although chemical manufacturers with a stake in the $16 billion BPA market continue to question this evidence, they’ve responded to safety concerns by offering BPA-free alternatives.

But as a recent study in *PLOS Genetics* [1] shows, the new versions seem an awful lot like the original. When one chemical comes under scrutiny, manufacturers often substitute compounds with similar structures to save time and money. But similar structures often cause similar problems. And that’s exactly what the *PLOS Genetics* authors found.

Thinking BPA’s replacement, bisphenol S (BPS), might target the same pathways, Patrick Allard and his colleagues at the University of California at Los Angeles compared the effects of both substances using the roundworm *Caenorhabditis elegans*, a favorite model for working out the genetics of meiosis, the process that creates sperm and oocytes.

The worms did not fare well. BPS, like BPA, wreaked havoc with their germ cells. Both compounds interfered with DNA damage repair as genetic material was exchanged during meiosis. That in turn triggered the death of developing germline cells and lowered fertility by producing fewer viable embryos. But, surprisingly, the authors say, the two bisphenols gummed up the works through somewhat different molecular steps.

That means simultaneous exposure to BPA and BPS could potentially cause even more reproductive harm. Future studies will need to confirm this possibility, but it’s an unsettling prospect given the ubiquity of BPA and increasing use of BPS, which has already been found in food, shampoo, face cream and other personal care products, soil, and thermal paper products.

And now there’s another “safer” alternative to worry about, researchers reported in *Environmental Health Perspectives* [2]. Suspecting that cashiers would face higher exposures on the job, the authors screened their urine, blood, and receipts (as well as people who didn’t handle receipts) for BPA, BPS, and another analog called BPSIP.

Cashiers’ BPA levels after work were highly variable, likely resulting from its widespread use, but levels of BPS and BPSIP were higher in most cases. Both BPS and BPSIP were also detected in people who weren’t cashiers, which was unexpected since researchers hadn’t found BPSIP in anything besides thermal paper before. The authors were also surprised to see BPSIP in cashiers’ blood more often than the other compounds, suggesting it may be more persistent and our exposure more widespread than previously assumed. BPSIP’s health effects are unknown.

Much more is known about BPA’s estrogenic powers. Earlier this year Pat Hunt, who was among the first to report BPA’s ability to scramble mouse eggs [3], reported similar problems in mouse sperm in *PLOS Genetics* [4].

Low sperm counts, undescended testicles, malformed penises, and other reproductive anomalies have risen in recent decades, suggesting environmental estrogens may play a role. Hunt’s team investigated this possibility by exposing newborn male mice to BPA or a stronger synthetic estrogen, ethinyl estradiol, just when sperm differentiation begins.

BPA exposure disrupts meiosis in males as it does in female mice, the authors discovered, but in different ways. In females, genetic exchange rates increase following BPA exposure, whereas in males they drop significantly. Errors during meiosis tended to kill developing sperm cells. But perhaps most surprising, and disturbing, these changes persisted. When unexposed animals received transplanted sperm stem cells from exposed males, they had the same problems.

Extra estrogen is not good for the prostate either. Normal prostate development proceeds as cells secrete precise levels of male and female hormones at carefully calibrated intervals. Health researchers are especially concerned about environmental contaminants that reach the womb during critical windows when even the slightest disturbance can rewire developmental programs to produce profound, irreversible changes that can take years to appear. These changes can cause a plethora of chronic health problems, including diabetes, cardiovascular disorders, birth defects, and cancer.

To see if BPA could help tip the balance toward prostate cancer, Esther Calderon-Gierszal and Gail Prins of the University of Illinois created a novel in vitro 3-D model of human prostate development to track the effects of BPA on differentiating human embryonic stem cells. The team reported in *PLOS ONE* [5] that BPA altered the growth pattern of the developing prostate and led to an abnormally expanded population of undifferentiated cells in the mature organ.

Mounting evidence over the past decade suggests that early exposures to environmental contaminants like BPA may be contributing to the obesity epidemic by disrupting energy homeostasis and metabolic programs [6]. This is not surprising since one of estrogen’s normal functions is to regulate fat cell differentiation and growth. Numerous studies have reported that exposing experimental animals to a range of contaminants, including BPA, DDT, dioxins, and phthalates, produces offspring that are more likely to accumulate fat and become obese [7]. Most evidence in humans has come from small samples that are difficult to interpret.

But last year Norwegian epidemiologists pooled data on thousands of European children to assess pre- and postnatal exposures to DDT and PCB, persistent environmental pollutants that were banned by nearly 200 countries decades ago. Babies with the highest concentrations of DDT metabolites in the womb (determined by analyzing cord blood) scored higher in growth measurements while with those with higher levels of PCB after birth (estimated in part by measuring the mother’s breast milk and blood) were smaller overall [8].

Aside from a suspected role in obesity, phthalates, a class of chemicals found in perfumes, deodorants, nail polish and numerous other consumer products, have also been linked to reproductive problems. In an *Environmental Health Perspectives* study [9] of women undergoing in vitro fertilization, the authors measured urine for several phthalate metabolites and found that women with higher DEHP and DiDP concentrations produced fewer eggs while those with higher DiNP and DiDP levels had fewer live births. This is not good news for women paying tens of thousands of dollars to conceive through IVF.

On the bright side, choosing products labeled “phthalate-free” can reduce your exposure to these chemicals [10].

The negative effects of estrogenic chemicals we encounter every day include an expanding list of changes to intricately choreographed programs that shape our capacity to grow and thrive. The complexity of these developmental dances, which overlap in space and time in the growing embryo, has often made it difficult for researchers to pin down exactly how these compounds cause harm. And that’s allowed chemical companies to continue arguing that there’s no evidence their products pose a threat to public health.

Yet Dodds warned over 50 years ago that continuous exposure to estrogens could pose serious health risks. He was worried about the long-term use of oral contraceptives, which were far stronger back then. Imagine what he would think of the constant stream of synthetic estrogens we’re exposed to today.

For more detailed reading on this subject, please see the associated PLOS Collection [11].
